# A new species of the genus *Capoeta* Valenciennes, 1842 from the Caspian Sea basin in Iran (Teleostei, Cyprinidae)

**DOI:** 10.3897/zookeys.682.12670

**Published:** 2017-07-05

**Authors:** Arash Jouladeh-Roudbar, Soheil Eagderi, Hamid Reza Ghanavi, Ignacio Doadrio

**Affiliations:** 1 Department of Fisheries, Faculty of Natural Resources, University of Tehran, Karaj, Alborz, Iran; 2 Department of Biology, Lund University, Lund, Sweden; 3 Biodiversity and Evolutionary Biology Department, Museo Nacional de Ciencias Naturales-CSIC, Madrid, Spain

**Keywords:** Algae-scraping cyprinid, Caspian Sea, inland freshwater, Iran, taxonomy

## Abstract

A new species of algae-scraping cyprinid of the genus *Capoeta* Valenciennes, 1842 is described from the Kheyroud River, located in the southern part of the Caspian Sea basin in Iran. The species differs from other members of this genus by a combination of the following characters: one pair of barbels; predorsal length equal to postdorsal length; maxillary barbel slightly smaller than eye’s horizontal diameter and reach to posterior margin of orbit; intranasal length slightly shorter than snout length; lateral line with 46–54 scales; 7–9 scales between dorsal-fin origin and lateral line, and 6–7 scales between anal-fin origin and lateral line.

## Introduction

Cyprinid fishes of the genus *Capoeta* Valenciennes, 1842 have a wide distribution throughout western Asia from Anatolia to the Levant, Transcaucasia, the Tigris and Euphrates basins, Turkmenistan, and northern Afghanistan (Bănărescu 1999; Levin et al. 2012; Ghanavi et al. 2016; Jouladeh-Roudbar et al. 2016). This genus has at least 28 species, of which the following 15 species are present in Iran: *Capoeta aculeata* (Valenciennes, 1844); *C. alborzensis* Jouladeh-Roudbar, Eagderi, Ghanavi & Doadrio, 2016; *C. anamisensis* Zareian, Esmaeili & Freyhof, 2016; *C. barroisi* Lortet, 1894; *C. buhsei* Kessler, 1877; *C. capoeta* (Güldenstaedt, 1773); *C. coadi* Alwan, Zareian, & Esmaeili, 2016; *C. damascina* (Valenciennes, 1842); *C. fusca* Nikolskii, 1897; *Capoeta gracilis* (Keyserling, 1861); *C. heratensis* (Keyserling, 1861); *C. mandica* Bianco & Bănărescu, 1982; *C. saadii* (Heckel, 1847), *C. trutta* (Heckel, 1843), and *C. umbla* (Heckel, 1843) (Jouladeh-Roudbar et al. 2015a,b; Alwan et al. 2016; Zareian et al. 2016; Jouladeh-Roudbar et al. 2016). Of these species, eight are endemic to Iran and three have been described recently based on the results of molecular studies (Alwan et al. 2016; Jouladeh-Roudbar et al. 2016; Zareian et al. 2016).

*Capoeta* species mainly inhabit fast flowing streams and rivers, but some species may also be found in lakes and springs (Turan et al. 2006). The members of this genus possess a fusiform body with small to moderately large scales and an inferior mouth (Coad 2017). Their lower lip bears a keratinized edge and lower lip is restricted to the corner of mouth (Howes 1982; Turan et al. 2006; Coad 2017). The dorsal fin is short with the last unbranched ray thickened, and has serrations posteriorly (serrations sometimes reduced to absent).

The populations of the genus *Capoeta* from the southern Caspian Sea basin are considered as belonging to two different species: *C. gracilis* and *C. capoeta* (Esmaeili et al. 2010; Jouladeh-Roudbar et al. 2015b). *Capoeta gracilis* was originally described from rivers near Esfahan, central Iran (Esfahan basin) and *C. capoeta* from Tiflis (Caspian Sea basin), Georgia (the Caspian Sea basin) (Güldenstädt 1773; Temminck and Schlegel 1843; Coad 2017). Several authors have considered *C. gracilis* as subspecies of *C. capoeta*, both with allopatric distribution. *Capoeta c. gracilis* was restricted to rivers between the Sefid and Atrak rivers in the southern part of the Caspian basin in Iran and *C. c. capoeta* to the Kura-Aras basin in western part of the Caspian basin (Bianco and Banarescu 1982). Furthermore, Bănărescu (1999) restricted the distribution of *C. c. gracilis* to the Urmia Lake basin and the Sefid River in southern part of the Caspian basin (and also to the lower Kura River in Azerbaijan) while C. capoeta aff. gracilis (an unnamed subspecies related to *C. c. gracilis*) was considered to inhabit the rest of the Iranian Caspian shore (Jouladeh-Roudbar et al. 2015). Posterior works have considered *C. gracilis* as a valid species but its distribution has been controversial (Esmaeili et al. 2014).

Currently, molecular studies have shown a high genetic differentiation in the populations of southern Caspian basins considered previously as *C. gracilis* or C. c. aff. gracilis and this led to the consideration of these populations as an undescribed species (Levin et al. 2012; Ghanavi et al. 2016). The presence of *C. capoeta* in both the Caspian Sea and Urmia Lake basins was also confirmed based on molecular and morphological data (Ghasemi et al. 2015; Ghanavi et al. 2016).

Previous phylogenetic and phylogeographic studies based on molecular mitochondrial data recognized three main clades within the genus *Capoeta*, Mesopotamian clade, Aralo-Caspian clade, and Anatolian-Iranian clade (Levin et al. 2012; Ghanavi et al. 2016). The Aralo-Caspian clade is composed by four valid species i.e. *C. capoeta*, *C. heratensis*, *C. fusca* and *C. alborzensis* in the Iranian freshwater basins (Ghanavi et al. 2016; Jouladeh-Roudbar et al. 2016). A detailed study of the populations of Aralo-Caspian clade in Iran, found some populations of the genus *Capoeta*, which were not identified as any described species (Ghanavi et al. 2016). Among them were populations distributed in the southern Caspian Sea basin, traditionally identified as *C. gracilis* (Jouladeh-Roudbar et al. 2015b). Our collection of the genus *Capoeta* from the southern Caspian Sea basin revealed the presence of two species, i.e. *C. capoeta* and an undescribed species (considered as *Capoeta* sp.1 in Ghanavi et al. 2016) that differ molecularly and morphologically from other described *Capoeta* species including species from the Esfahan basin (Alwan et al. 2016; Ghanavi et al. 2016). According to our intensive samplings from the Esfahan basin, only two species i.e. *C. aculeata* and *C. coadi* were found. Therefore, the main goal of this work is to study morphologically the populations of the collected *Capoeta* specimens from the southern Caspian Sea basin, north of Iran, previously assigned to *C. gracilis*, and to compare them with the remaining species of this genus from Iran, and based on differences found, they are described as a new species herein.

## Materials and methods

**Figure 1.**
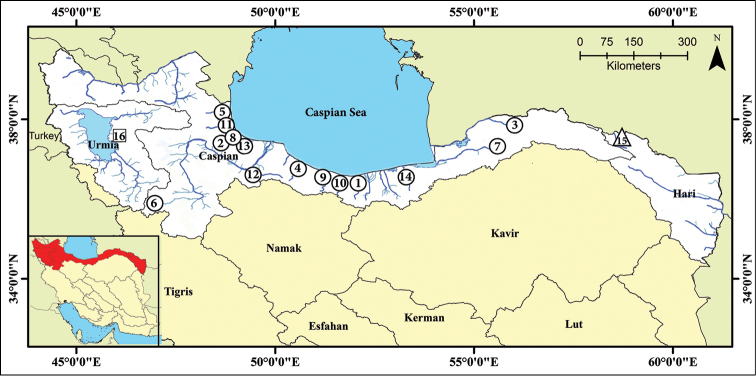
Map of the southern Caspian Sea basin and sampling points. Numbers of the sampling sites correspond to the numbers of sampling sites in Table 1, circle: *Capoeta razii* sp. n., triangle: *C. fusca*, square: *C. capoeta*.

Approximately 150 specimens of the genus *Capoeta* were collected by electrofishing at 14 sites covering most of its distribution area in southern Caspian Basin (Figure 1, Table 1). Fin clips stored in 96% ethanol and deposited in the Tissue and DNA Collection of the Ichtyological Museum of Natural Resources Faculty – University of Tehran (IMNRF-UT). The fish were killed with overdoses of MS222, were fixed in 10% formalin, and were later preserved in the Ichthyology collection of IMNRF-UT, Iran. For morphometric purposes and to have a base for molecular studies 23 individuals of *C. capoeta* and *C. fusca* from the Urmia Lake and Hari River basins, respectively, were also analysed.

**Table 1.** Sampling sites and coordinates. Numbers in the first column (Loc) correspond to numbers on the sampling map in Figure 1.

Loc.	River	Locality	Species	GPS Coordinates	Alt. (m)
1	Angueta Rud	Sangetab	*Capoeta razii* sp. n.	36°28'37"N, 42°13'31"E	44
2	Asalem	Asalem		37°42'53"N, 48°55'44"E	104
3	Atrak	Maraveh Tappeh		37°54'30"N, 55°57'10"E	198
4	Chalk Rud	Katalom		36°52'19"N, 50°46'17"E	-20
5	Choobar Rud	Choobar		38°10'36"N, 48°52'54"E	-7
6	Ghezel Ozan	Nesareh		35°52'12"N, 47°04'54"E	1732
7	Golestan	Tangrah		37°22'55"N, 55°51'12"E	564
8	Karrgan Rud	Talesh		37°48'02"N, 48°53'04"E	71
9	Kelar Abad Rud	Kelar Abad		36°42'05"N, 51°13'10"E	-15
10	Kheyr Rud	Chalos		36°36'35"N, 51°33'45"E	34
11	Khushavar Rud	Khushavar		38°01'51"N, 48°53'31"E	17
12	Sefid Rud	Lowshan		36°38'13."N, 49°29'17"E	307
13	Shafa Rud	Punel		37°31'52"N, 49°06'36"E	246
14	Tajan	Payin Hular (Sari)		36°29'12"N, 53°05'10"E	90
15	Ghale Chay	Ajab Shir	*C. capoeta*	37°29'25"N, 45°59'57"E	
16	Segonbadan	Farooj	*C. fusca*	37°14'46"N, 58°08'01"E	

**Morphological examinations.** Thirty morphometric measurements and thirteen meristic character countings were performed using a digital caliper to the nearest 0.1 mm and stereomicroscope, respectively (Tables 4–8). Measurements follow Kottelat and Freyhof (2007). Fin ray counts separate unbranched and branched rays. The last two branched rays articulated on a single pterygiophore in dorsal and anal-fins are noted as “1”.

An allometric method was used to remove size-dependent variation in morphometric characters using following formula (Elliott et al. 1995): M_adj_ = M(L_s_/L_0_)*^b^*, where M is the original measurement, M_adj_ the size adjusted measurement, L_0_ the standard length of the fish, L_s_ the overall mean of the standard length for all fish from all samples in each analysis, and *b* was estimated for each character from the observed data as the slope of the regression of log M on log L_0_ using all fish in any group. The adjusted morphometric characters of the studied populations were analysed using Principal Component Analysis (PCA) and compared by Non-Parametric Multivariate Analysis of Variance (NPMANOVA) based on the *P*-values obtained from permutation test with 1000 replicates in PAST software (version 2.14). The meristic characters of the studied populations were analysed using Correspondence Analysis (CA), and compared by Non-Parametric Multivariate Analysis Of Variance (NPMANOVA) based on the Bonferoni-corrected *P*-values obtained from permutation test with 1000 replicates in PAST software (version 2.14).

**Molecular data analysis.** To analyse the molecular composition we studied the complete mitochondrial cytochrome *b* gene of all species of Aralo-Caspian group which include an unnamed population from Caspian Sea basin (Levin et al. 2012; Ghanavi et al. 2016). In this study, we considered sequences obtained from previous studies and deposited in GenBank (Table 2) (Levin et al. 2012; Ghanavi et al. 2016; Zareian et al. 2016; Jouladeh-Roudbar et al. 2016). Sequences were aligned using Geneious software (Geneious v. 10.0.2, Biomatters, http://www.geneious.com/), and visually verified to maximize positional homology. Sequences of *Luciobarbus capito* (Güldenstädt, 1773), *L. brachycephalus* (Kessler, 1872) and *L. subquincunciatus* (Günther, 1868) species were chosen as outgroup based on their phylogenetic relationship to genus *Capoeta* (Levin et al. 2012; Yang et al. 2015; Ghanavi et al. 2016). Uncorrected pairwise genetic distances (p-distances) between species (Table 3) were calculated with Mega 6 (Tamura et al. 2013). A bootstrapping process was implemented with 1000 repetitions. Jmodeltest 2.1.4 (Darriba et al. 2012) selected TrN+I as the best evolutionary model. RAxML (Stamatakis 2006) implemented in GENEIOUS software was used to estimate the maximum-likelihood (ML) tree. Bayesian inference was conducted with MrBAYES v. 3.2.2 (Ronquist et al. 2012). Two simultaneous analyses were run on 2*10^7^ generations, each with four MCMC chains sampling tree every 2000 generations. Convergence was checked on Tracer 1.6 (Rambaut and Drummond 2013). After discarding the first 10% of generations as burn-in, we obtained the 50% majority rule consensus tree and the posterior probabilities. The species delimitation methodology used was Bayesian Poisson tree process (bPTP) model which is based on a distance-based tree (Zhang et al. 2013). bPTP were accessed at Exelixis Labs (http://sco.h-its.org/exelixis/web/software/PTP/index.html). Haplotype genealogies were visualized by HaploView v. 4.2 (Barrett et al. 2005).

**Table 2.** List of species used for molecular analysis for *Cyt b* and GenBank accession number.

KU312380	*Capoeta anamisensis*	KU167903	*Capoeta razii* sp. n.	JF798266	*Capoeta aculeata*
KU312381		KU167905		KM459640	
JF798279	*Capoeta barroisi*	KM459627		KM459638	
KM459651	*Capoeta mandica*	KM459628		KM459637	
KM459649		KU167933		JF798267	
KM459650		KM459630		KM459631	*Capoeta saadii*
AF145949	*Capoeta trutta*	KU167922		KM459639	
KM459673		KU167934		KM459641	
JF798332		KU167932		KU167952	*Capoeta damascina*
KU167893	*Capoeta heratensis*	KU167913		KU167953	
JF798317		KU167911		KU167954	
JF798318		KU167912		KM459624	*Capoeta buhsei*
JF798319		KU167918		KM459623	
JF798316		KM459696	*Capoeta alborzensis*	JF798283	
KU167894		KY365754		KM459634	*Capoeta coadi*
KU167936	*Capoeta capoeta*	KY365752		JF798285	
KU167937		KY365753		KM459633	
KU167938		KM459695		AF145937	*Luciobarbus subquincunciatus*
KU312371	*Capoeta fusca*	KM459688		KP712171	*Luciobarbus capito*
KU312372		KM459687		AY004729	*Luciobarbus brachycephalus*

**Table 3.** Estimates of evolutionary divergence over sequence pairs between *Capoeta razii* sp. n. and other Iranian *Capoeta* species.

	species	1	2	3	4	5	6	7	8	9	10	11	12	13	14	15	16
1	*L. subquincunciatus*	–															
2	*L. capito*	9.5	–														
3	*L. brachycephalus*	8.6	3.3	–													
4	*C. barroisi*	8.6	9.0	8.6	–												
5	*C. trutta*	9.7	9.3	9.2	1.2	–											
6	*C. mandica*	9.6	9.0	8.7	1.3	1.1	–										
7	*C. anamisensis*	9.2	8.4	8.6	1.6	1.4	1.5	–									
8	*C. saadii*	9.8	8.7	9.0	7.6	7.9	8.0	8.2	–								
9	*C. damascina*	9.2	8.3	8.8	7.5	7.9	7.9	8.3	2.8	–							
10	*C. buhsei*	9.6	8.6	9.3	8.1	8.4	8.4	8.7	2.6	2.2	–						
11	*C. coadi*	9.6	8.6	9.4	7.8	8.1	8.0	8.7	2.7	2.1	1.4	–					
12	*C. fusca*	8.8	8.9	8.5	8.5	8.9	9.1	8.9	6.5	6.4	5.7	6.3	–				
13	*C. alborzensis*	8.7	8.2	8.6	7.9	8.3	8.5	8.3	5.6	5.4	5.3	5.5	1.6	–			
14	*C. aculeata*	9.3	8.8	8.8	8.2	8.6	8.8	8.7	6.1	5.9	5.9	6.0	2.2	1.3	–		
15	*C. heratensis*	10.1	9.1	9.7	9.1	9.3	9.5	9.0	6.2	5.9	5.8	6.5	2.5	2.2	2.6	–	
16	*C. capoeta*	9.0	8.5	8.6	7.9	8.4	8.6	7.9	5.9	5.6	5.8	5.9	2.3	1.8	2.0	2.6	–
17	*C. razii* sp. n.	9.5	9.1	9.3	8.4	8.8	9.1	8.8	6.0	5.8	5.8	5.9	2.2	1.4	1.8	2.5	2.1

### Abbreviations

**SL** standard length;

**HL** lateral head length;

**IMNRFI-UT** Ichtyological Museum of Natural Resources Faculty.

## Results

**Figure 2.**
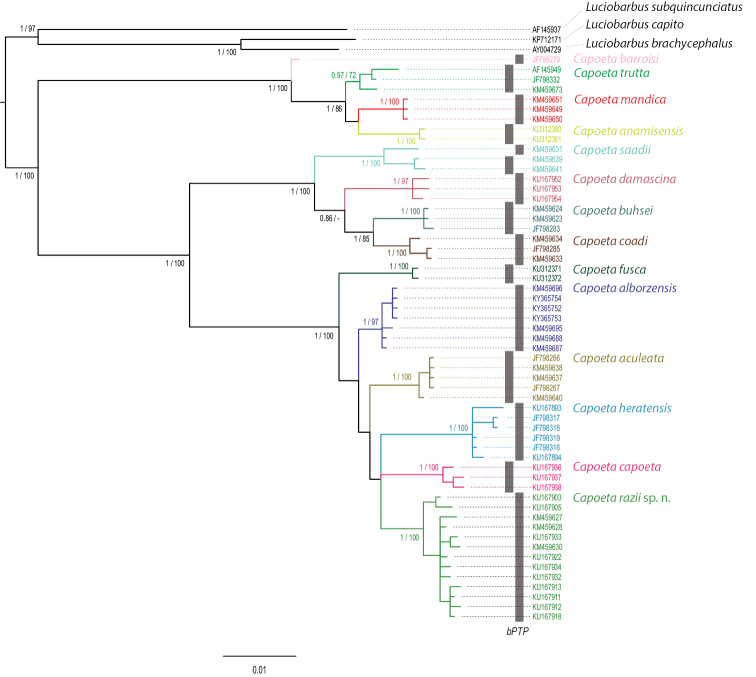
*Capoeta* genus; Values at nodes correspond to BI posterior probability/ML bootstrap. Grey bars represent the species delimitations performed with bPTP software.

**Figure 3.**
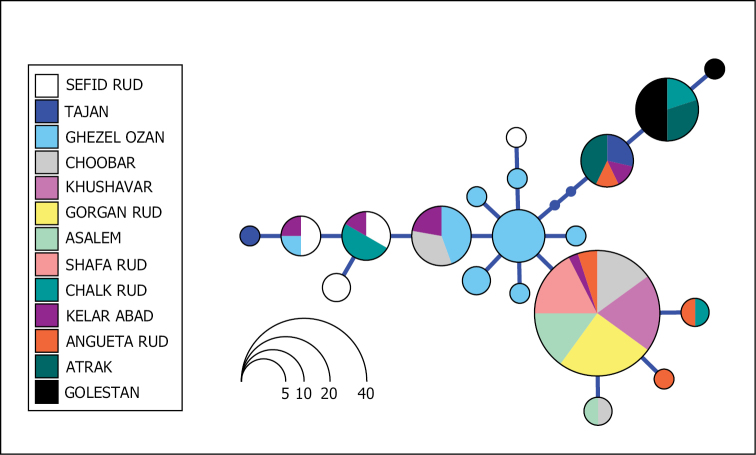
Haplotype networks of available specimens of Caspian Sea basin. Each independent river system is represented by a different colour. Data from Ghanavi et al. 2016.

Based on the results, from the 1040 bp of complete mitochondrial cytochrome *b* genes, 793 positions were conserved and 195 were parsimony informative. Genetic distances between species are listed in Table 3. The Bayesian and ML analyses yielded similar topologies with well-supported nodes (Figure 2). The reconstructed topology was also in agreement with previously published higher-level phylogenies that included *Capoeta* and the three main clades, Aralo-Caspian, Anatolian-Iranian, and Mesopotamian were recovered (Levin et al. 2012; Ghanavi et al. 2016; Jouladeh-Roudbar et al. 2016). Based on molecular phylogeny, the differentiation of populations from Caspian Sea basin from the other described species is shown. The species delimitation methodology also supports these populations to be considered as a different species from the other populations included in the study (Figure 2). The haplotype network does not show any geographical patterns between the different populations of the suggested species in the closely located but independent rivers of the Caspian Sea basin (Figure 3).

The result of PCA analysis showed that all specimens explained 45.79% of morphometric variations by the first two PC axes extracted from the variance-covariance matrix (PC1=27.60% and PC2=18.19%). Plotting of first and second PCs displayed a complete segregation of the three populations. In addition, NPMANOVA showed significant differences between all studied populations in terms of the morphometric characters (P<0.001) (Figure 11). The result of CA showed that all specimens explained 63.1% of morphometric variations by the first two CA (PCA1=35.82% and CA2=27.28%). Plotting of first and second CAs displayed a complete segregation of the three populations. In addition, NPMANOVA showed significant differences between all studied populations in terms of the morphometric characters (P<0.0001) (Figure 12).

### Capoeta razii

Taxon classificationAnimaliaCypriniformesCyprinidae

http://zoobank.org/948BD913-A0DF-4371-97F6-B707CE56CFD6

**Figure 4.**
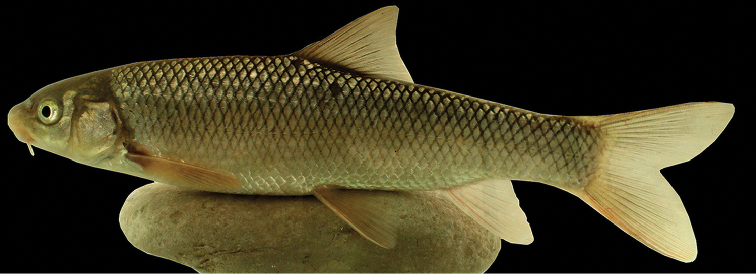
*Capoeta razii* sp. n., IMNRF-UT-1072-9, holotype, SL: 142.6 mm, Iran: Mazandaran Prov., Chalos city, Kheyroud River, Caspian Sea basin.

**Figure 5.**
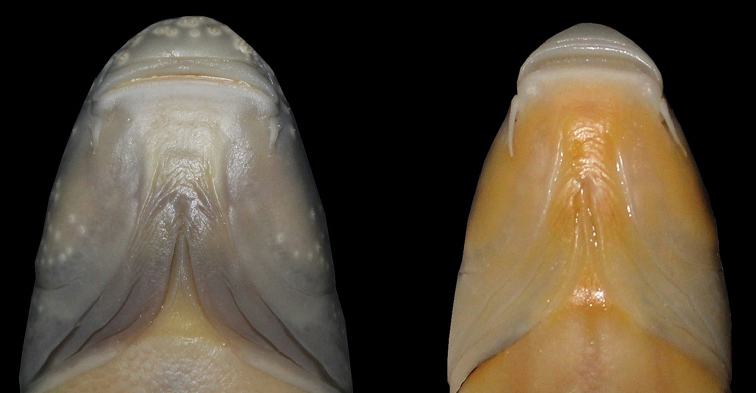
Ventral view of Head. *Capoeta razii* sp. n. (right, IMNRF-UT-1072-11, SL: 109 mm) and *C. capoeta* (left, IMNRF-UT-1067-6, SL: 110 mm).

**Figure 6.**
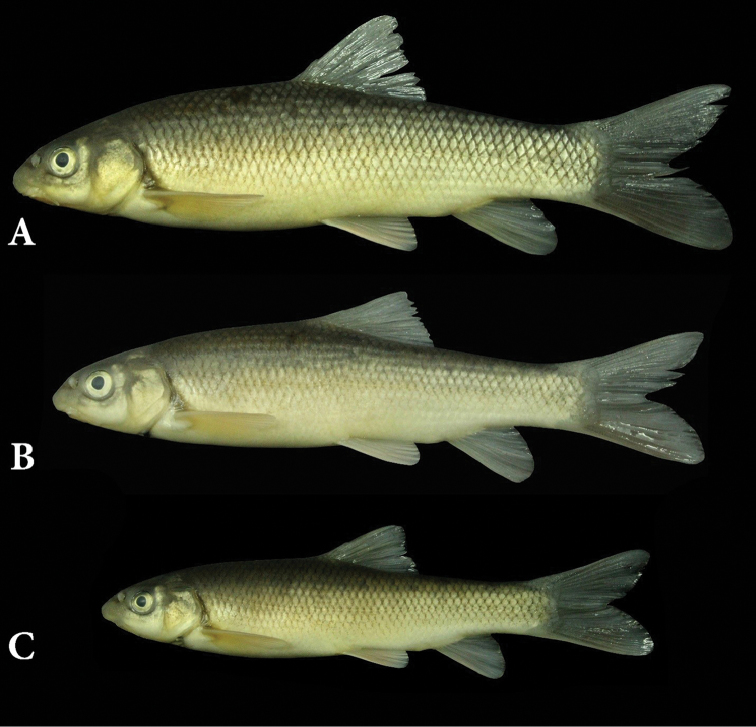
*Capoeta razii* sp. n., paratypes; **A** IMNRF-UT-4, SL: 130 mm **B** IMNRF-UT-12, SL: 115 mm **C** IMNRF-UT-3, SL: 99 mm.

**Figure 7.**
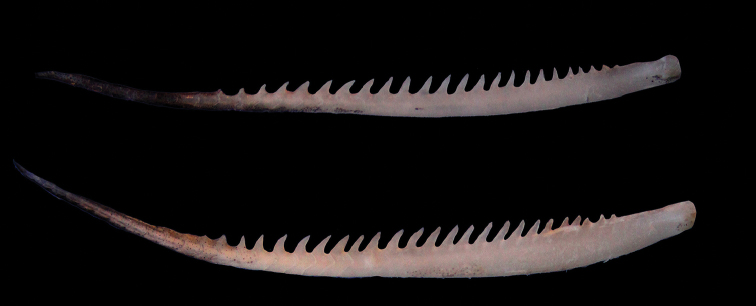
Last simple dorsal-fin rays, *Capoeta razii* sp. n. (Below, IMNRF-UT-1066-9, SL: 116) and *C. capoeta* (Above, IMNRF-UT-1067-13, SL: 121 mm).

Figures 4 , 5 , 6 , 7

#### Holotype.

**Figure 8.**
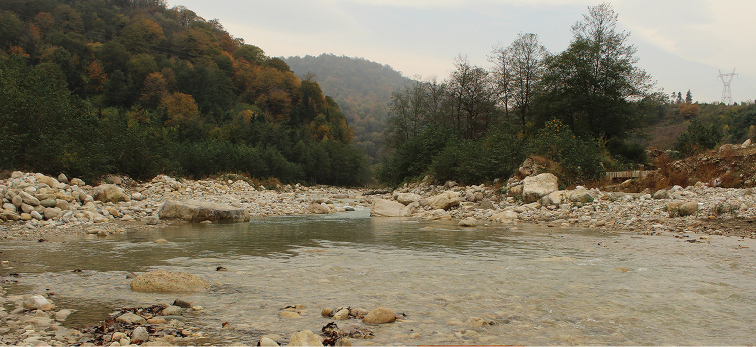
Kheyroud River, near Chalos city, Caspian Sea basin, type locality of *Capoeta razii* sp. n.

IMNRF-UT-1072-9, holotype, 142.6 mm SL. Iran: Mazandaran Prov., Chalus city, Kheyroud River (Figure 8), Caspian Sea basin, 36°36'35"N, 51°33'45"E, S. Eagderi & A. Jouladeh-Roudbar, November 2016.

#### Paratypes.

IMNRF-UT-1072, 14 specimens, 90.7–184.2 mm SL; data same as holotype.

#### Diagnosis.

*Capoeta razii* sp. n. is distinguished from the other species of *Capoeta* in Iran by a following combination of characters, none of them unique. One pair of barbels; pre-dorsal length equal to postdorsal length; maxillary barbel slightly smaller than eye’s horizontal diameter and reach to posterior margin of orbit; intranasal length slightly shorter than snout length; lateral line with 46–54 scales, 7–9 scales between dorsal-fin origin and lateral line and 6–7 scales between anal-fin origin and lateral line.

**Table 4.** Morphometric data of *Capoeta razii* sp. n. (holotype, IMNRF-UT-1072-9; paratypes, IMNRF-1072, 14 specimens) *C. capoeta* (IMNRF-UT-1067, 15 specimens) and *C. fusca* (IMNRF-UT-1065, 8 specimens).

Characters	Holotype	*C. razii* sp. n.			*C. capoeta*			*C. fusca*		
		Range	Mean	SD	Range	Mean	SD	Range	Mean	SD
Standard length (mm)	142.6	90.7–184.2			66.5–157.3			47.2–124.2		
**In percent of standard length (SL)**										
Body depth maximal	23.7	23.1–25.5	23.9	0.7	23.4–26.9	25.2	1.0	24.4–27.1	26.0	0.9
Caudal peduncle depth	12.1	11.1–12.9	11.9	0.5	10.1–12.6	11.7	0.7	11.1–13.5	12.5	0.8
Predorsal length	52.3	50.2–53.1	51.8	0.9	50.8–55.5	52.9	1.2	52.6–55.0	53.8	0.9
Postdorsal length	51.8	49.9–54.2	51.7	1.2	47.6–55.1	51.9	2.1	48.9–52.3	50.6	1.2
Prepelvic length	55.1	55–58.7	56.1	1.1	54.3–61.3	57.1	1.9	55.2–58.6	57.3	1.2
Preanal length	75.9	76.4–79.6	77.6	1.0	74.9–79.7	77.5	1.4	76.7–79.9	78.4	1.3
Caudal peduncle length	18.9	16.1–19.4	17.4	1.1	14.7–20.0	17.2	1.4	14.2–17.9	16.1	1.3
Dorsal fin base length	11.3	12.1–15.4	13.6	0.9	12.7–16.7	14.5	1.4	14.9–18.0	16.5	0.9
Dorsal fin depth	17.7	16.2–21	18.9	1.2	18.5–22.2	20.5	0.9	18.7–26.1	22.3	2.2
Anal fin base length	7.3	6.8–8.3	7.5	0.4	6.0–9.1	7.7	0.8	8.1–10.1	9.1	0.7
Anal fin depth	16.8	15–20.4	17.7	1.4	14.4–18	16.2	1.0	17.1–19.9	18.7	0.8
Pectoral fin length	20.5	17.8–21.3	19.5	1.1	15.4–20.6	18.7	1.9	18.3–24.2	21.2	2.1
Pelvic fin length	16.7	14.1–17.5	16.0	1.0	14.2–17.3	16.0	0.9	15.9–19.9	18.1	1.2
Pectoral – pelvic-fin origin distance	32.3	30.6–36.1	32.8	1.4	31.4–37.0	34.2	1.7	29.5–34.5	32.3	1.8
Pelvic – anal-fin origin distance	20.6	21–24.2	22.2	1.0	18.7–23.0	21.5	1.2	20.1–23.9	22.1	1.4
Body width	16.3	15.1–17	16.0	0.6	16.3–18.4	17.2	0.6	16.6–18.7	17.6	0.7
Caudal peduncle width	3.6	2.8–4.1	3.4	0.5	3.1–4.2	3.7	0.3	5.5–7.0	6.3	0.5
Head length (HL)	22.5	20.5–24	23.0	1.0	19.8–25.9	22.6	1.8	25.0–28.6	26.2	1.7
**As percentage of head length (HL)**										
Snout length	26.2	26.2–31.6	28.7	1.4	24.7–29.8	27.1	1.6	28.2–33.1	30.6	1.9
Eye horizontal diameter	20.1	17.1–26.7	23.3	2.7	17.4–22.7	19.4	1.7	15.4–23.7	19.3	2.9
Postorbital distance	53.5	46.4–54.4	50.7	2.2	47.9–60.8	56.2	3.4	48.1–54.2	52.2	2.0
Head depth at nape	78.3	70.1–82.9	76.4	3.5	67.5–87.5	79.4	5.2	70.3–76.1	72.6	2.0
Head depth at eye	50.2	45.7–53	51.1	2.0	44.8–56.8	52.7	3.2	47.0–53.4	51.2	1.9
Head length at nape	90.1	88.9–97	92.2	2.4	83.8–98.6	92.9	3.9	87.9–96.3	91.5	3.1
Head width	67.6	61.6–73.1	65.9	3.1	62.3–77.3	70.0	5.4	54.9–69.7	60.7	4.7
Inter orbital	42.5	34.3–46	42.8	2.9	41.4–52.2	46.2	3.4	35.7–40.1	37.0	1.4
Inter nasal	26.1	20.2–26	24.7	1.8	24.0–31.3	28.0	2.2	17.1–23.6	20.7	1.8
Mouth width	35.6	28.7–37.9	34.2	2.9	31.4–41.3	36.0	2.9	26.6–38.9	31.3	4.7
Barbel length	13.0	14–21.6	17.2	2.4	9.3–16.2	13.2	1.8	9.9–17.3	13.6	2.9

**Table 5.** Number of scales above lateral line (ALL), below lateral line (BLL), Number Dorsal Soft Rays (DSR)/Hard (DHR), Anal Soft Rays (ASR)/Anal Hard Rays (AHR), pelvic (PLR) fin rays and Number Gill rakers on the lower limb (LOL) in *Capoeta razii* sp. n. and *C. capoeta*.

Species	3	4	5	6	7	8	9	10	Mod	Mean	SD
**ALL**											
*Capoeta razii* sp. n.					3	10	2		8	7.9	0.6
*Capoeta capoeta*						3	10	2	9	8.9	0.6
**BLL**											
*Capoeta razii* sp. n.				10	5				6	6.3	0.5
*Capoeta capoeta*					12	3			7	7.2	0.4
**DHR**											
*Capoeta razii* sp. n.	1	14							4	3.9	0.3
*Capoeta capoeta*	7	8							4	3.6	0.5
**DSR**											
*Capoeta razii* sp. n.					2	13			8	7.9	0.4
*Capoeta capoeta*					3	12			8	7.8	0.4
**AHR**											
*Capoeta razii* sp. n.	15								3	3.0	0.0
*Capoeta capoeta*	15								3	3.0	0.0
**ASR**											
*Capoeta razii* sp. n.				15					6	6.0	0.0
*Capoeta capoeta*				15					6	6.0	0.0
**PLR**											
*Capoeta razii* sp. n.						1	10	4	9	9.2	0.6
*C. capoeta*							9	6	9	9.3	0.6
**LOL**											
*Capoeta razii* sp. n.		4	12	1					5	4.9	0.5
*Capoeta capoeta*		2	11	2					5	5.0	0.5

**Table 6.** Number of pectoral (PFR), caudal fin rays (DFR), total gill rakers (TGR) and circum-pendicular scales (CPS) in *Capoeta razii* sp. n. and *C. capoeta*.

Species	15	16	17	18	19	20	21	Mod	Mean	SD
**PFR**										
*Capoeta razii* sp. n.		2	7	2	1			17	17.4	1.1
*Capoeta capoeta*				6	5	2	2	18	18.9	1.3
**CFR**										
*Capoeta razii* sp. n.				1	14			19	18.9	0.3
*Capoeta capoeta*					10	5		19	19.3	0.5
**CPS**										
*Capoeta razii* sp. n.			6	9				18	17.6	0.5
*Capoeta capoeta*				10	3	2		18	18.5	0.7
**TGR**										
*Capoeta razii* sp. n.	1		2	8	2	1	1	18	18.1	1.4
*Capoeta capoeta*					2	6	7	21	20.3	0.7

**Table 7.** Number of total lateral-line scales in *Capoeta razii* sp. n. and *C. capoeta*.

Species			Total lateral line Scales											Mod	Mean	SD
	46	47	48	49	50	51	52	53	54	55	56	57	58			
*Capoeta razii* sp. n.	2	1	4	2	2	-	2	-	2					48	49.1	2.3
*Capoeta capoeta*										4	5	4	2	56	56.3	1.0

**Table 8.** Range of meristic features of Iranian *Capoeta* species.

No.	Species	LL	ALL	BLL	CPS	TGR	Reference
1	*Capoeta alborzensis*	39–44	6–8	5–8	16–17	19–22	This study
2	*Capoeta aculeata*	39–43	7–8	5–7	16–20	19–23	This study
3	*Capoeta razii* sp. n.	46–54	7–9	6–7	17–18	15–21	This study
4	*Capoeta anamisensis*	56–67	11–12	6–8	–	21–25	Zareian et al. 2016
5	*Capoeta barroisi*	76–84	14–16	10–13	–	26–29	Turan et al. 2006
6	*Capoeta buhsei*	80–89	13–15	11–13	29–31	11–13	This study
7	*Capoeta capoeta*	51–58	9–11	7–8	19–23	17–29	This study
8	*Capoeta coadi*	68–75	12–15	9–10	25–29	15–18	This study
9	*Capoeta damascina*	64–82	12–17	8–12	23–30	17–25	Alwan, 2011
10	*Capoeta fusca*	46–54	8–10	8–9	19–26	16–18	This study
11	*Capoeta heratensis*	55–61	9–12	7–9	22–25	21–24	This study
12	*Capoeta mandica*	58–68	12–13	8–10	27–33	23–27	Alwan et al. 2016
13	*Capoeta saadi*	61–78	9–14	6–10	–	12–17	Alwan, 2011
14	*Capoeta trutta*	65–82	9–14	9–12	27–31	20–30	This study
15	*Capoeta umbla*	90–102	18–23	12–14	33–36	18–20	This study

#### Description.

See Figure 4 for general appearance and Tables 4–7 for morphometric and meristic data. Body is moderately deepened and compressed laterally. Greatest body depth occurs at the level of dorsal-fin origin. Dorsal profile of the head is convex. Predorsal length is equal to post-dorsal length. Dorsal profile of the body is convex without any keel in the front of dorsal-fin origin. Snout is rounded with a triangular view in ventral. Mouth is almost straight. Upper and lower lips are adnate to jaws. Lower jaw has a strong keratinized edge. Rostral cap is well developed and usually overlaps with upper lip. One set of maxillary barbels that are short, slightly smaller than eye’s horizontal diameter, reaching to posterior margin of orbit. Intranasal length is slightly shorter than snout length. Pelvic axillary scales are triangular, well developed, and pointed. Dorsal fin has 3–4 unbranched and 7–8 branched rays, its outer margin is straight or slightly concave. Last unbranched dorsal-fin ray is thickened and serrated, distally flexible, and with 15–25 serrae on its posterior margin, with serrations along 50–70% of its posterior margin, denticles are long and narrowly spaced but not strongly developed. Last unbranched dorsal-fin ray slightly shorter than first branched ray, and the tip is soft. Pelvic fins are inserted under posterior of the first branched dorsal-fin base. Caudal fin is deeply forked with pointed and equal size of lobes. Pectoral fin has 16–19 branched rays. Pelvic fin has 1 unbranched and 9–10 branched rays. Anal fin has 2–3 unbranched rays, 6 branched rays and its outer margin is usually convex or straight. There are 15–21 gill rakers on the outer side of the first arch. There are 17–18 circum-peduncular scales. Lateral line is complete, with 46–54 scales. There are 7–9 scales between the dorsal-fin origin and lateral line and 6–7 are located between the anal-fin origin and lateral line.

#### Colouration.

In life, the upper part of the body is golden brown, olive-green, or silver, and the belly is whitish up to the lateral line. The head is dark-brown or olive-green on top and the cheeks are pale brown to white (Figure 4). Anal, pelvic, and pectoral fins are hyaline or light brown, and dorsal and caudal fins have a narrow black line on rays. In specimen smaller than 50 mm SL, minute black spots are present on flanks.

When preserved, the dorsum is dark brown on back and flanks, and yellowish white on belly (Figure 6). Dorsum of the head is dark brown, and the cheeks beige. Fins are often light brown and pelvic and anal fins may be yellowish to hyaline. Dorsal and caudal fins are darker than lower fins. Peritoneum is black.

#### Distribution and habitat.

*Capoeta razii* is found in many rivers and streams of the southern Caspian Sea basin. It is one of the most abundant species in the Caspian Sea basin along with the members of the genus *Alburnoides* Jeitteles, 1861. At the Kheyroud River (type locality), the current was medium to fast, river width was between 3–14 m and the maximum depth was around one meter, the stream bed was composed of cobbles and gravel, and the riparian vegetation type was deciduous forests. Following fish species: *Poticola iranicus* Vasil’eva, Mousavi-Sabet & Vasil’ev 2015, *Alburnoides taberstanensis* Mousavi-Sabet, Anvarifar & Azizi, 2015, *Alburnus chalcoides* (Güldenstädt 1772), *Barbus cyri* De Filippi 1865, *Squalius turcicus* De Filippi 1865, *Luciobarbus capito* Güldenstädt 1773, *L. mursa* Güldenstädt 1773, *Cobitis faridpaki* Mousavi-Sabet, Vasil’eva, Vatandoust & Vasil’ev 2011, co-exist with *C. razii* in type locality. *Capoeta razii* is known from most of rivers and streams between Atrak and Kote komeh (Near Astara city) rivers in southern Caspian Sea basin.

#### Etymology.

The new species is named in honour of Abū Bakr Muhammad ibn Zakariyyā al-Rāzī, a Persian polymath, physician, alchemist, and philosopher, for his important contributions in the history of medicine. He also discovered numerous compounds including Ethanol.

#### Remarks.

*Capoeta razii* sp. n. is distinguished from *C. aculeata* and *C. alborzensis* by a smaller scale size and a higher number of total lateral line scales (46–54 vs. 39–44).

*Capoeta razii* sp. n. is distinguished from *C. fusca*, by a smaller caudal peduncle width (2.8–4.1 vs. 5.5–7.0 %SL), a smaller head length (20.5–24.0 vs. 25.0–28.6 %SL), and the presence of numerous minute scales on the caudal fin base extending distally onto the fin membranes for more than half the fin ray length (vs. absence of minute scales on the caudal fin base) (Figure 10).

*Capoeta razii* sp. n. is distinguished from *C. anamisensis*, *C. barroisi*, *C. buhsei*, *C. Capoeta*, *C. coadi*, *C. damascina*, *C. heratensis*, *C. mandica*, *C. saadi* and *C. umbla* by a larger scale size, a fewer number of total lateral line scales (46–54 vs. 55–102).

**Figure 9.**
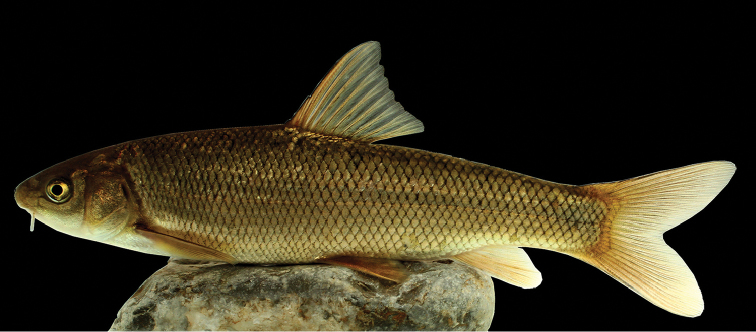
Uncatalogued live specimen of *Capoeta capoeta*. Iran: Ajab Shir town, Ghale Chay River, Urmia basin.

**Figure 10.**
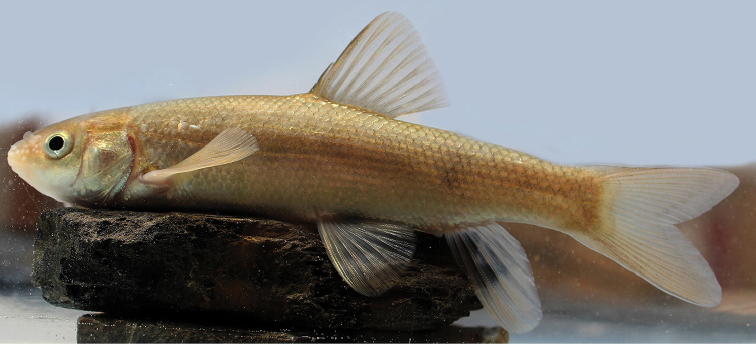
Live specimen of *Capoeta fusca*, IMNRF-UT-1065-1, SL: 124 mm, Iran: North Khorasan prov.: Near Farooj town, at segonbadan village, Qanat-e Segonbadan, Hari basin.

#### Comparative material.

– *Capoeta aculeata*: IMNRF-UT-1058, 9. 53–116 mm SL, Iran: Fars prov.: Tange Boragh village, Kor River, Kor basin, 37°14'46"N, 58°08'01"E, Aug 2014, S. Eagderi & H. Mossavi-Sabet. – *Capoeta alborzensis*.: IMNRF-1063, 7. 50–153mm SL, Iran: Tehran prov.: Nam River, tributary of Hableh River, near Arjomand village, 35°48'00"N, 52°30'57"E; IMNRF-UT-2063, 23, 46–163mm SL, Iran: Tehran prov.: Nam River, tributary of Hableh River, Kavir basin, near Harandeh village, 35°42'41"N, 52°40'19"E, S. Eagderi & A. Jouladeh-Roudbar, September 2014. – *Capoeta buhsei*: IMNRF-UT-1075, 12. 103.9–211.8 mm SL, Iran: Markazi prov.: Tafresh town, at Khalife kandy village, Mazlaghan Chay River, Namak basin, 34°45'34"N, 49°56'50"E, Nov 2016, A. Rahmani, M. A. Jahazi, R. Rahbar-zare, A. Jouladeh-Roudbar. – *Capoeta capoeta*: IMNRF-UT-1067, 15. 66–157 mm SL, Iran: Tabriz prov.: Near Ajab shir city, Ghale Chay River, Urmia Lake basin, 37°29'25"N, 45°59'57"E, Nov 2016, T. Hosseinpour, M. Ahmadian & A. Jouladeh-Roudbar. – *Capoeta coadi*: IMNRF-UT- 1074, 15. 125.7–194.7 mm SL, Iran: Chaharmahal and Bakhtiari prov.: Near Joneghan town, at Darkesh varkesh village, Behesht Abad River, Tigris basin, 32°05'22"N, 50°39'54"E, Aug 2016, T. Hosseinpour, A. Soleymani & A. Jouladeh-Roudbar. – *Capoeta fusca*: IMNRF-UT-1065, 8. 46–121 mm SL, Iran: North Khorasan prov.: Near Farooj town, at segonbadan village, Qanat-e Segonbadan, Hari basin, 37°14'46"N, 58°08'01"E, Jun 2016, S. Eagderi & A. Jouladeh-Roudbar. – *Capoeta heratensis*: IMNRF-UT-1064, 15. 116–161 mm SL, Iran: Khorasan-e Razavi prov.: Near Sarakhs, at Pole-e Khaton, Hari River, Hari basin, 35°56'51"N, 61°08'51"E, Jun 2016, S. Eagderi & A. Jouladeh-Roudbar. – *Capoeta trutta*: IMNRF-UT- 1073, 15. 54.1–165.2 mm SL, Iran: Kermanshah prov.: Songhor to Satar road, Tape Esmail village, Gavehroud River, Tigris basin, 34°56'01"N, 47°12'49"E, Aug 2016, T. Hosseinpour, A. Soleymani & A. Jouladeh-Roudbar. – *Capoeta umbla*: IMNRF-UT-1077, 15. 107.3–175.9 mm SL, Iran: Kurdistan prov.: Near Sardasht town, Barisu village, Little Zab River, Tigris, 36°08'48"N, 45°32'17"E, May 2016, S. Eagderi, H. Porbagher, P. Jalili & A. Jouladeh-Roudbar.

**Figure 11.**
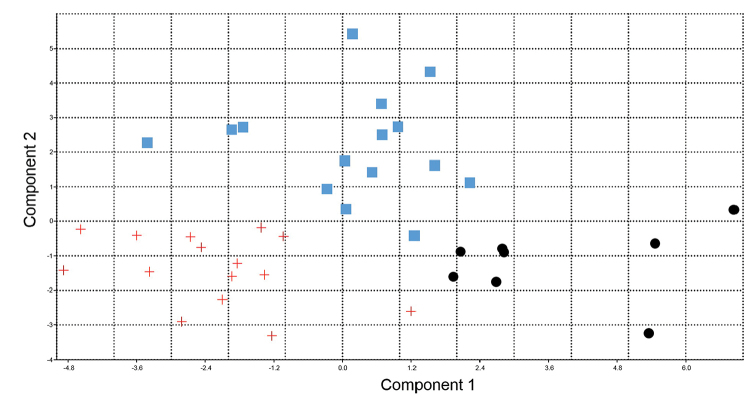
Principal component analysis of relative morphometric characters of the *Capoeta razii* sp. n. (+) *C. fusca* (•) and *C. capoeta* (◾) populations.

**Figure 12.**
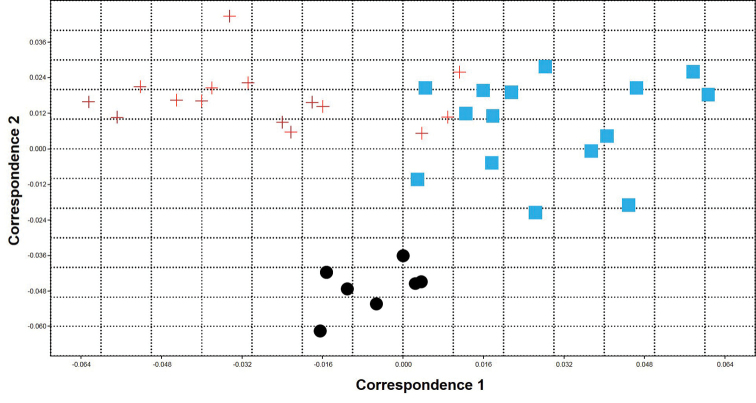
Correspondence analysis of meristic characters of the *Capoeta razii* sp. n. (+) *C. fusca* (•) and *C. capoeta* (◾) populations.

## Supplementary Material

XML Treatment for Capoeta razii
